# mHealth Innovation for Aging With Mobility Disability

**DOI:** 10.1093/geroni/igaf122.241

**Published:** 2025-12-31

**Authors:** Elena Remillard, Eric Levitan

## Abstract

The landscape of telehealth has changed drastically over the last decade, extending beyond the remote delivery of clinical services (i.e., telemedicine). Worldwide, there has been continued growth in the popularity of mHealth (mobile health) applications to support physical, mental, and social health. From virtual exercise classes to remote health monitoring systems, mHealth can utilize a variety of mobile technologies such as smart phones, laptops, and wearable devices. mHealth applications can be especially beneficial for people aging with mobility disabilities who experience transportation and accessibility barriers with in-person programs. This session will highlight innovative mHealth solutions from the Rehabilitation Engineering Research Center on Technologies to Support Aging among People with Long-Term Disabilities (RERC TechSAge). Mitzner et al. will present results from the TechSAge Tele Tai Chi clinical trial – an evidence-based tai chi program delivered via Zoom designed to be socially engaging and inclusive of older adults with mobility limitations. Rice et al. will describe the development of a falls detection and monitoring system for older adults who use wheelchairs that integrates smart watch monitoring and a user-facing app. Hsieh et al., will highlight the design a fall risk mHealth app for people with Multiple sclerosis (MS) that enables users to measure and evaluate their fall risk and engage in preventative strategies. Eric Levitan, CEO & Founder of Vivo, an online, live fitness program designed for older adults, will serve as the discussant, engaging participants in interactive discussion and offering an industry perspective on challenges implementing mHealth applications for older adults.

